# Development and validation of a self-efficacy questionnaire (SE-12) measuring the clinical communication skills of health care professionals

**DOI:** 10.1186/s12909-016-0798-7

**Published:** 2016-10-18

**Authors:** Mette K. Axboe, Kaj S. Christensen, Poul-Erik Kofoed, Jette Ammentorp

**Affiliations:** 1 Health Services Research Unit, Lillebaelt Hospital, and Institute of Regional Health Research, University of Southern Denmark, Kabbeltoft 25, 7100 Vejle, Denmark; 2 Research Unit for General Practice, Department of Public Health, Aarhus University, Bartholins Allé 2, 8000 Aarhus, Denmark; 3 Department of Paediatrics, Lillebaelt Hospital, and Institute of Regional Health Research, University of Southern Denmark, Skovvangen, 6000 Kolding, Denmark

**Keywords:** Communication skills training, Self-efficacy, Self-assessment, Calgary-Cambridge Guide, Questionnaire, Validity, Reliability

## Abstract

**Background:**

The outcome of communication training is widely measured by self-efficacy ratings, and different questionnaires have been used. Nevertheless, none of these questionnaires have been formally validated through systematic measurement of assessment properties. Consequently, we decided to further develop a self-efficacy questionnaire which has been used in previous studies. This study aims to examine the content, internal structure, and relations with other variables of the new version of the self-efficacy questionnaire (SE-12).

**Methods:**

The questionnaire was developed on the basis of the theoretical approach applied in the communication course, statements from former course participants, teachers, and experts in the field. The questionnaire was initially validated through face-to-face interviews with 9 staff members following a test-retest including 195 participants.

**Results:**

After minor adjustments, the SE-12 questionnaire demonstrated evidence of content validity. An explorative factor analysis indicated unidimensionality with highly correlated items. A Cronbach’s α of 0.95 and a Loevinger’s H coefficient of 0.71 provided evidence of statistical reliability and scalability. The test-retest reliability had a value of 0.71 when evaluated using intra-class correlation. Expected relations with other variables were partially confirmed in two of three hypotheses, but a ceiling effect was present in 9 of 12 items.

**Conclusions:**

The SE-12 scale should be regarded a reliable and partially valid instrument. We consider the questionnaire useful for self-evaluation of clinical communication skills; the SE-12 is user-friendly and can be administered as an electronic questionnaire. However, future research should explore potential needs for adjustments to reduce the identified ceiling effect.

**Electronic supplementary material:**

The online version of this article (doi:10.1186/s12909-016-0798-7) contains supplementary material, which is available to authorized users.

## Background

A training course is a well-known and reliable method to enhance the communication skills among clinicians and thereby ensure better interaction with patients. Several studies have shown that the clinical communicative behavior of clinicians can be improved after participating in a communication training course [1–4]. Clinical communication skills are not just a personal trait; it is a series of modifiable skills that can be developed to become a better communicator [5]. Effective clinical communication that improves accuracy and efficiency has been shown to have a positive impact on several aspects of patient outcome, such as patient satisfaction, adherence, symptom relief, and physiological outcome [5].

The effects of a patient-centered communication skills training course have been tested in a randomized controlled trial and in a pre-post intervention study at Lillebaelt Hospital in Denmark [6, 7]. Both studies showed significant improvements in the clinicians’ self-efficacy after course participation. These positive results have led to the implementation of a 3-day communication training course for the entire clinical staff of approximately 2,500 people. The course was developed by the Danish Medical Association and was inspired by the practical guidelines and scientific publications of British psychiatrist Peter Maguire [4, 8]. The course is based on the communication skills described in the Calgary-Cambridge Guide, which defines a curriculum of 71 clinical communication skills [5]. The course utilizes multiple teaching tools including role-playing, dialogues, and video recordings with patients.

To evaluate the impact of the training course, we decided to use self-efficacy rating as a measurement tool. Self-efficacy is a widely used construct for self-assessment of the outcome of communication skills training [6, 9–13]. The psychologist Albert Bandura defines self-efficacy as a person’s own belief in his or her ability to perform a specified task successfully. Self-efficacy concerns a person’s judgment of what s/he can accomplish with own skill set [14], i.e. what s/he believes that s/he can do. Therefore, self-efficacy is believed to have a direct influence on personal performance in specific contexts [15]. Changes in behaviour can occur as a result of learning, experience, and feedback [15, 16].

Positive correlations between communication skills training and increased levels of self-efficacy have previously been documented [7, 9, 11–13]. However, self-reported assessment of self-efficacy has been criticized for its lack of accuracy compared to objective assessments [17, 18]. Nevertheless, a recent study showed no statistically significant differences between the self-efficacy scores reported for communication skills in a group of medical students, scores reported by observers, and scores based on patient assessment of the same skills [19].

Evaluating the impact of communication skills training of 2,500 clinical staff members called for a method that was cost-effective and time-saving as opposed to objective rating methods. Different questionnaires have been used to evaluate clinicians’ self-efficacy in communication, but many lack formal validation with appropriate measurement properties [1, 13, 20]. Research has shown that the impact of a certain training course can be assessed by an instrument closely tailored to the curriculum being taught [21]. Consequently, we needed a tool which included key elements from the Calgary-Cambridge Guide. We further developed the self-efficacy questionnaire that we had used in previous studies [6, 7] by gradually adjusting and improving the questionnaire until its final version. The included items thus reflect the tasks and objectives within the structure of the Calgary-Cambridge Guide: initiating the session, gathering information, providing structure to the consultation, building the relationship, explaining and planning, and closing the session [5]. Although the guide was originally developed for medical interviews performed by physicians, studies have shown that it can also be useful and effective among other medical clinicians, such as nurses [6, 7, 9]. Therefore, the aim of this study was to provide evidence for the validity of this instrument in terms of content, internal structure and relations with other variables.

## Methods

### Construction of the questionnaire

We intended to create a generic assessment instrument to capture the skills used in prolonged patient-centered conversations performed by the different occupational groups, primarily physicians, nurses, health care assistants, midwives, physiotherapists, and occupational therapists. It was also essential to design a questionnaire capable of measuring the clinicians’ self-efficacy both before and after attending the communication skills training course to compare the level of skills evaluated by perceived self-efficacy. The target population was essential in the selection of items for the questionnaire. Communication teachers and former course participants were included in focus group discussions to provide a good framework for SE item construction. After some adjustments in consideration of the population of interest, we selected twelve questions reflecting general clinical communication skills. Each question began with the words: “How certain are you that you are able to successfully …” followed by a specific communication skill. A 10-point response scale ranging from 1 (very uncertain) to 10 (very certain) was chosen inspired by Bandura’s guide for constructing self-efficacy scales [22]. Although Bandura recommends a 0–10 response scale, we chose to use a 1–10 scale and add a “not relevant” check box. Respondents were advised to use this check box only if s/he could not find a specific item/communication skill relevant for their clinical practice. In addition to the 12 self-efficacy items, the questionnaire contained 5 items regarding background data about the course participants.

### Data collection

The data collection process consisted of two phases:

1. A content validation study, including qualitative data from interviews with 9 participants and qualitative data obtained from comments in the questionnaire used in the test-retest study.
2. A questionnaire study, including responses from 787 clinicians affiliated with four departments at three different hospitals; 292 responded to the initial questionnaire and 195 responded to both the first and the second questionnaire.

### Evidence of content validity

A content validation was conducted to examine the relevance, coverage, and understandability of the items as experienced by test participants [23, 24]. The informants were a representative set of diverse professional backgrounds, gender, and age. In addition, participants were asked if they had any comments on the 12 self-efficacy questions in the questionnaire or had anything to add regarding the subject.

### Evidence of internal structure

With the exception of reliability, the following measurement properties are based on data from the 292 clinicians who completed the initial questionnaire. Reliability is based on the responses from the 195 clinicians who completed both the first and second questionnaire and answered *no change* to the anchor question.

### Dimension of data

Identification of dimensionality is especially important when interpreting the scoring of items. Within a given dimension, scores can be summarized and collectively expressed for the trend. Factor analysis is a well-known method for examining how many significant dimensions can be recognized in the dataset. Items that are highly correlated are clustered to one factor, whereas items within a single factor will have low correlation with items associated to other factors [25]. We performed an explorative factor analysis to study the number of dimensions present in our dataset.

### Internal consistency

Internal consistency concerns the interrelatedness of the items in a questionnaire scale and how well the items measure the same construct [24]. Cronbach’s α is considered an adequate measure of internal consistency provided thatthe scale is considered unidimensional. A low Cronbach’s α indicates lack of correlation between items in a scale. A very high Cronbach’s α (>0.95) implies high correlation among the items in the scale, which may indicate redundancy of one or more items [26]. We used a cut-off point of 0.7, which is widely accepted for a Cronbach’s α [24, 27].

In addition, we performed a Mokken scale analysis (MSA) to determine if the items were ranked. MSA is based on the principles of item response theory, which originates from the Guttman scale and the assumptions of cumulativity of item responses. In MSA, Loevinger’s H computes the ratio between observed and expected error rates for each pair of items between a given item and all other items in a scale or among all possible pairs of items in the scale. A Loevinger’s H > 0.50 indicates good scalability [28].

### Reliability

A measure of reliability is the degree to which systematic measurement errors are absent in the measurement results. A test-retest procedure is one way to evaluate the reliability of results across different sampling sets [23, 24, 26]. In this study, the reliability was calculated by completing the questionnaire on two occasions. We used intra-class correlation coefficient (ICC) [24] for continuous measures as a parameter for reliability.

### Test-retest reliability

The minimum acceptable level of test-retest reliability was set at a value of 0.70. The purpose of conducting a test-retest was to assess the reproducibility of the data and to determine the degree to which repeated measurements (test-retest) provide similar answers under steady conditions [23, 24]. Clinicians, with the exclusion of those who only had minimal patient contact, from four different departments participated in the test-retest: oncology; gastrointestinal surgery; and two orthopaedic departments. Two of the four departments had formerly participated in the communication skills training course due to executive decisions within these independently administered departments. We strived to include at least 10 respondents per item in the questionnaire, which is considered adequate for assessing measurement characteristics [24]. The first questionnaire was initially mailed to 787 clinicians who received an e-mail with a link to the web-based questionnaire. Answering all questions was mandatory. The interval between the test and the retest was approximately two weeks, which was considered short enough to prevent changes in the clinicians’ communication skills and long enough to prevent recollection of the previous responses given. To address the stability of questionnaire results, an anchor question was added in the second questionnaire: “*In comparison to the first time you answered the questionnaire, do you believe that your communication skills have changed according to the skills adressed*”. Table 1 displays the demographic data of the participants who completed both the first and the second assessment and evaluated their communication skills to be unchanged between the first and the second assessment.

**Table 1:** Demographic data of participants in the test-retest (*N = 195*). Distribution of gender, profession, age, and former experience with communication training in the four participating departments and in total

	Trained group^a^		Not trained group		
Department	Gastrointestinal *n* = 34	Orthopedics *n* = 64	Oncology *n* = 75	Orthopedics *n* = 22	Total *n* = 195
Gender					
Male n (%)	4 (11.8)	11 (17.2)	6 (8.0)	13 (59.1)	34 (17.4)
Female	30 (88.2)	53 (82.8)	69 (92.0)	9 (40.9)	161 (82.6)
Age groups					
Mean (range) (y)	45 (28–61)	45 (26–62)	47 (25–59)	45 (27–65)	46 (25–65)
Profession, n (%)					
Physician, specialist	4 (11.76)	12 (18.75)	4 (5.33)	9 (40.91)	29 (14.9)
Physician, non-specialist	2 (5.88)	3 (4.69)	9 (12.00)	5 (22.73)	19 (9.7)
Nurses	25 (73.53)	47 (73.44)	56 (74.67)	7 (31.82)	135 (69.2)
Nursing assistants	2 (5.88)	0 (0.00)	5 (6.67)	1 (4.55)	8 (4.1)
Others	1 (2.94)	2 (3.13)	1 (1.33)	0 (0.00)	4 (2.1)
Previously participated in communication courses n (%)	29 (85.3)	52 (81.3)	31 (41.3)	10 (45.5)	122 (62.6)

^a^Departments that previously participated in the communication skills training course conducted by The Danish Medical Association

### Evidence of validity based on relations with other variables

The construct validity refers to the extent to which scores on a particular instrument relate to other measures in a way that is consistent with the hypotheses concerning the construct that is being measured [24, 29]. In the absence of a gold we assessed the construct validity by formulating three hypotheses based on previous findings in similar settings [6, 7, 11].

1. We should observe higher self-efficacy scores for clinicians from the two departments that previously participated in the communications skills training course compared to the two departments that did not.
2. We should observe higher self-efficacy scores for clinicians with long employment experience in their current department compared to clinicians with less experience.
3. We should observe the highest self-efficacy scores among physicians, followed by nurses, and lowest among nursing assistants.

Within all three hypotheses, self-efficacy scores were measured as the sum of responses across the 12 measured communication skills.

### Floor and ceiling effects

The presence of floor or ceiling effects may indicate that extreme response items are missing in the lower or upper end of the scale. Changes are thus difficult to measure as some respondents may have achieved the lowest or highest score the first time they completed the questionnaire, which tends to result in limited responsiveness [29]. Floor or ceiling effects were considered to be present if >15 % of the respondents achieved the lowest or highest possible score, respectively [30].

### Data management

The data was analysed using Stata (v. 12.1) and ICC with SPSS statistical software (v. 17.0).

### Ethical considerations

An expert committee at the Faculty of Health Sciences, University of Southern Denmark, which is responsible for ensuring that both scientific and ethical considerations are in compliance with the Declaration of Helsinki, approved both the study design and the protocol. Permission to obtain and keep records including name and contact information of clinicians was granted by the Danish Data Protection Agency.

For the interviews conducted, verbal consent to participate was obtained from all participants. All heads of department involved gave permission for their staff to take part in the test-retest. All participants were informed of the purpose of the study and assured that all collected data would be treated anonymously to ensure that participating individuals could not be identified.

## Results

### Content validity

All of the participants in the qualitative test of the questionnaire considered the 12 self-efficacy items to be relevant. Additionally, none of the participants commented upon areas lacking in the questionnaire. Participants were generally pleased with the response scale because it resembled scales used in their daily routines with patients. Suggestions for minor adjustments in the phrasing of a couple of questions were made, and the wording was changed accordingly. We also received a few comments regarding the questionnaire in the test-retest. The comments mainly addressed the last part of item four concerning *change of focus*. Some participants found it difficult not to change focus if the conversation with the patient was “heading in the wrong direction”. Therefore, this item was shortened, which also removed ambiguity due to conjoined questions (Additional file 1).

### Test-retest

We received completed questionnaires from 292 of 787 surveyed staff members, giving a response rate of 37 %. A total of 195 of the 787 (25 %) staff members responded to both questionnaires and rated their communication skills as stable. Table 2 displays the distribution of answers in total and between the two departments which had previously participated in the course) and the two departments which had not participated in the course).

**Table 2:** Descriptive statistics of the 12 self-efficacy items (range, 1–10). Distribution of answers according to group, gender, age, profession (nurses and nursing assistants merged), seniority, and respondents marking highest possible score. The trained group had formerly participated in the communication skills training course conducted by The Danish Medical Association as opposed to the not trained group who had not participated in the communication skills training course

Item How certain are you that you are able to successfully …	Mean total (SD)	Trained group (SD)	Not trained group (SD)	Gender Male (*n* = 34) Female (*n* = 161)	Age (year) 21- (*n* = 14) 31- (*n* = 52) 41- (*n* = 67) 51- (*n* = 58) 61- (*n* = 4)	Profession Physicians (*n* = 47) Nurses (*n* = 145) Others (*n* = 3)	Seniority (year) < ½ (*n* = 17) ½-1 (*n* = 5) 1–2 (*n* = 15) 2–5 (*n* = 61) 5–10 (*n* = 46) >10 (*n* = 51)	Respondents marking highest possible score %
1: …identify the issues the patient wishes to address during the conversation?	8.07 (1.34)	8.21 (1.44)	7.93 (1.23)	8.39 8.01	6.79 7.89 8.28 8.23 9.50	8.04 8.12 6.75	7.12 8.50 7.73 8.03 8.33 8.27	14^b^
2: …make an agenda/plan for the conversation with the patient?	7.89 (1.59)	8.22 (1.55)	7.64 (1.60)	8.39 7.88	6.31 7.87 8.23 8.06 9.25	8.04 8.00 6.00	6.47 8.25 8.23 7.96 8.11 8.27	15^b^
3: …urge the patient to expand on his or her problems/worries?	8.39 (1.37)	8.47 (1.48)	8.31 (1.26)	8.35 8.39	7.43 8.22 8.46 8.62 9.75	8.26 8.47 7.00	7.53 8.75 8.13 8.32 8.51 8.69	21
4: ^a^ …listen attentively without interrupting or changing of focus?	8.41 (1.43)	8.51 (1.42)	8.37 (1.45)	8.19 8.50	8.21 8.20 8.49 8.63 9.50	8.09 8.60 7.50	7.00 9.25 8.40 8.36 8.60 8.88	21
5: …encourage the patient to express thoughts and feelings?	8.27 (1.47)	8.33 (1.67)	8.28 (1.26)	8.06 8.36	7.93 7.98 8.48 8.45 9.50	7.98 8.47 6.50	7.41 9.00 7.87 8.08 8.42 8.88	16^b^
6: …structure the conversation with the patient?	8.05 (1.44)	8.24 (1.40)	7.88 (1.46)	8.52 7.99	6.86 8.00 8.29 8.13 9.25	8.32 8.05 6.25	7.06 8.75 7.87 8.07 8.17 8.37	14^b^
7: …demonstrate appropriate non-verbal behavior (*eye contact, facial expression, placement, posture, and voicing*)?	8.58 (1.18)	8.71 (1.21)	8.45 (1.14)	8.52 8.59	8.64 8.54 8.54 8.60 9.25	8.47 8.62 8.50	8.35 8.75 8.73 8.42 8.69 8.67	23
8: …show empathy (*acknowledge the patient’s views and feelings*)?	8.87 (1.01)	8.92 (1.08)	8.83 (0.93)	8.90 8.87	9.00 8.83 8.91 8.79 9.50	8.79 8.91 8.75	8.71 8.75 9.07 8.81 8.89 8.94	28
9: …clarify what the patient knows in order to communicate the right amount of information?	8.35 (1.22)	8.44 (1.25)	8.26 (1.20)	8.45 8.33	7.64 8.11 8.53 8.49 9.25	8.28 8.42 6.75	7.44 8.50 8.13 8.37 8.46 8.57	16^b^
10: …check patient’s understanding of the information given?	8.45 (1.25)	8.62 (1.24)	8.32 (1.24)	8.42 8.48	7.86 8.19 8.68 8.60 9.25	8.32 8.55 7.25	7.76 8.75 8.40 8.32 8.54 8.82	19^b^
11: …make a plan based on shared decisions between you and the patient?	8.49 (1.24)	8.61 (1.24)	8.37 (1.24)	8.81 8.43	7.79 8.39 8.59 8.59 9.50	8.66 8.47 7.25	7.82 8.75 8.53 8.35 8.60 8.76	19^b^
12: …close the conversation by assuring, that the patient’s questions have been answered?	8.79 (1.18)	8.84 (1.20)	8.60 (1.16)	9.10 8.65	8.07 8.74 8.77 8.77 9.25	8.81 8.68 9.00	8.18 9.00 8.87 8.69 8.83 8.78	27
Sum score (mean)	100.61 (8.38)	102.12 (8.51)	99.24 (8.27)					

^a^After the test-retest question no. 4 was changed to: “*How certain are you that you are able to successfully listen attentively to the patient?”*

^b^These questions in group 2 did not exceed the >15 % set as a limit for the ceiling effect

The questionnaire was translated and back-translated with the purpose of presenting the items in this paper. Only the Danish version has been tested accordingly to the described procedure

The “not relevant” check box was used 57 times across the 12 items, which accounted for 2.4 % of the answers given.

### Dimensionality of data

**Fig. 1:**
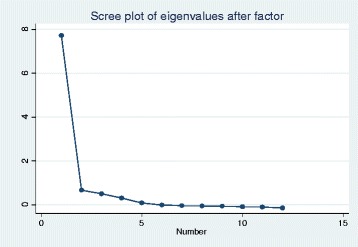
Screeplot of eigenvalues according to factors. One factor is accounting for 87.7 % responses of the SE-12 in principal factor analysis

An explorative factor analysis was performed using a principal factor method with oblique rotation. The result, which was based on examination of eigenvalues, loadings, and screen plots, showed a single dominant factor, indicating that the 12 self-efficacy items correlated highly with each other (Figure 1). The scale is, therefore, unidimensional, which allows summarization of items. The same result was noted in the oblique (Varimax) and Promax rotations with the factor loading cut-off value set at ≥ 0.4.

### Internal consistency

The internal consistency in the 12 self-efficacy questions was high with a Cronbach’s α of 0.95 (range, 0.94–0.95), which indicates high correlations among the items in the scale. In the Mokken Analysis, the Loevinger’s H turned out to be high, with a total scale coefficient of 0.71 (range, 0.63–0.75). This suggests that the items were rank-ordered, with no substantial overlap of items and, therefore, additive.

### Relations with the validity of other variables

#### Hypothesis 1

When comparing the sum scores in group 1 with those of group 2, we found higher scores in all the self-efficacy questions in group 1, i.e. the two departments with staff who had previously participated in the course.

The mean sum score in group 1 (*n* = 152) was 101.27 (SD = 15.84), whereas the mean sum score in group 2 (*n* = 140) was 96.99 (SD = 13.5). The t-test resulted in t = 2.47 (*P* = 0.01), which confirmed our hypothesis.

#### Hypothesis 2

Participants with the most experience within their field had a higher self-efficacy sum score compared to participants with less experience. A Kruskal-Wallis equality-of-populations rank test was performed (chi-square = 12.94 with 5 degrees of freedom; *P* = 0.024). This finding confirmed our expectation that self-efficacy is highly correlated to experience in the field.

#### Hypothesis 3

The difference in self-efficacy sum scores between professions showed that nurses had a higher mean sum score (mean = 100.20, SD = 15.08) than physicians (mean = 98.80, SD = 12.33), although the difference was not statistically significant (t = 0.72, *P* = 0.47). After adjusting for length of service, physicians had higher self-efficacy sum scores, but the result was still not statistically significant. Nurses had higher self-efficacy sum scores (mean = 100.20, SD = 15.08) compared to nursing assistants (mean = 93.42, SD = 20.42), but the difference was, again, not statistically significant (t = 1.81, *P* = 0.07). Our results did neither support nor reject the hypothesis that we should observe the highest self-efficacy scores among physicians, followed by nurses, and lowest among nursing assistants physicians, nurses, and nursing assistants.

### Test-retest reliability

The test-retest reliability was acceptable for the entire self-efficacy scale, with an ICC agreement of 0.71 (0.66–0.76). A higher reliability was observed in the two departments with clinicians who had previously participated in the communication course (*n* = 98), with an ICC agreement of 0.77 (range, 0.67 – 0.84). Furthermore, fair to good reliability was found in the two departments with staff who had not previously attended the communication course (*n* = 97), with an ICC agreement of 0.64 (range, 0.49 – 0.79).

### Floor and ceiling effects

A ceiling effect was present in 9 of 12 self-efficacy questions, which exceeds the >15 % set as a limit. The distribution of respondents marking the highest possible score is shown in Table 2. Despite the presence of a ceiling effect, we did not change the scale as similar questionnaires in comparable settings have successfully detected changes in self-efficacy in study participants after receiving communication skills training [6, 7, 11]. None of the self-efficacy questions exceeded >15 % in the floor effect.

## Discussion

The findings from this study showed that the SE-12 questionnaire is a unidimensional, reliable, and partially valid instrument for assessment of clinicians’ self-efficacy in clinical communication before and after receiving communication skills training in the current context.

SE-12 was found to be comprehensive and easy to understand in our content validity test. However, one item was shortened in accordance with the comments received in the test-retest. Inclusion of more participants during the face-validity test might have enabled us to discover this shortcoming at an earlier stage.

The internal consistency of the SE-12 scale was at the higher end of the acceptable range, which resulted in an elevated risk of redundant items. It might be valuable to test if Cronbach’s α would decrease if one or more of the items were deleted. Because the SE-12 questionnaire is already short and fairly easy and quick to complete, we did not reduce the number of items. Instead, we performed a Mokken scale analysis, which confirmed that no item reduction was necessary because Loevinger’s H coefficients were high, which suggests rank ordering and cumulative distribution of the 12 items.

To determine the relations with the validity of other variables, we tested three hypotheses. We anticipated an increase in self-efficacy scores among the staff from group 1, including the two departments that had previously participated in the communication course conducted by the Danish Medical Association. As expected, a significantly higher score was found in group 1 compared to group 2. This difference would most likely have been even greater if the staff members from group 2 had had less experience and practice from other communication courses. Surprisingly, more than 40 % of the staff members from group 2 had one day or more of training in communication skills; this training was provided by other parties than the Danish Medical Association, but it had overlapping curriculum. Despite this unexpected slightly higher level of communication training in group 2 and the fact that staff members in group 1 being were from surgical departments, the SE-12 questionnaire was still capable of detecting a difference between these two groups.

Our results are similar to previous studies performed in similar settings, although a slightly different self-efficacy questionnaire was used [6, 7]. We did not achieve statistically significant differences in the ranking of self-efficacy scores among the different occupational groups. After adjusting for seniority, our results showed that physicians tend to have higher self-efficacy in their clinical communication skills than nurses and nursing assistants. However, the groups of physicians and nursing assistants were considerable smaller than the group of nurses and, therefore, not likely to show significance after adjustment for seniority. In fact, our study showed to be underpowered for formal testing of this particular hypothesis.

When determining the reliability of the test-retest, we found an acceptable ICC of 0.71, which is just above our cut-off value of 0.70. We believe that our result is unrelated to variations in the communication skills of our participants; our findings are more likely to be associated with our response scale. It can be discussed whether inclusion of the “not relevant” check categoryis pertinent in the questionnaire, given that so few made use of it. Nevertheless, we did not remove this option because we wanted every respondent to have the opportunity to submit all answers as desired, especially because it was mandatory to answer all the items in the questionnaire.

We believe that our validation process was robust and transparent and that it allows others in different settings to test for correlations with the SE-12 questionnaire. Nonetheless, a ceiling effect was present, which might impact the responsiveness and the interpretability of the questionnaire. It also leaves limited room for detecting improvements in each individual participant. However, when looking only at the respondents who had not participated in the communication skills training course, 7 out of 12 items were not affected by the ceiling effect (Table 2). This tells us that, to some degree, we are able to detect an improvement in self-efficacy after course participation compared to baseline. Still, the presence of a ceiling effect indicates that the SE-12 questionnaire needs further testing with an adjusted response scale or that minor modifications of the questions are required. Alternatively, if practitioners wish to use the questionnaire in its current state and rank respondents in the upper end of the scale, the Tobit model might be a useful tool for analysing the data. The Tobit model is capable of correcting inference when ceiling effect is present by using a variation of multiple regression [31].

## Conclusions

The SE-12 questionnaire has some adequate measurement qualities for the assessment of clinicians’ self-efficacy in the context of clinical communication skills training. The questionnaire is user-friendly and can easily be administered as an electronic questionnaire. The questionnaire measured one single dominant factor, presumably self-efficacy. We found acceptable reliability (ICC: 0.71), which indicated absence of systematic errors in the measurements, and high correlation between the items in the scale (Cronbach’s α:, 0.95). However, we only identified partial relations with the validity of other variables by confirming two out of the three constructed hypotheses regarding clinical communication skills. We acknowledge that the existing ceiling effect is an issue that needs further attention, either by testing alternative response scales or by using the Tobit model to check for potential presence of ceiling effect.

## Additional file

Additional file 1: SE-12, english version.(PDF 217 kb)
