# Supplementary material for: Development and validation of a self-efficacy questionnaire (SE-12) measuring the clinical communication skills of health care professionals
Source: BMC Med Educ. 2016 Oct 18;16:272. doi: 10.1186/s12909-016-0798-7 (PMC5069791; doi:10.1186/s12909-016-0798-7)
Supplement: Additional file 1: — SE-12, english version.(PDF 217 kb) [file 12909_2016_798_MOESM1_ESM.pdf]

# Communication with the patient

**The following questions concern selected communication skills regarding the conversation with the patient. Please answer every question, and only use the 'not relevant' box if the question asked does not apply to you in your daily work.**

On a scale from 1-10, 1 = very uncertain 10 = very certain:

**1 How certain are you that you are able to successfully identify the issues the patient wishes to address during the conversation?**

| Very uncertain | | | | | | | | | Very certain | Not relevant |
|---|---|---|---|---|---|---|---|---|---|---|
| 1 ☐ | 2 ☐ | 3 ☐ | 4 ☐ | 5 ☐ | 6 ☐ | 7 ☐ | 8 ☐ | 9 ☐ | 10 ☐ | ☐ |

**2 How certain are you that you are able to successfully make an agenda/plan for the conversation with the patient?**

| Very uncertain | | | | | | | | | Very certain | Not relevant |
|---|---|---|---|---|---|---|---|---|---|---|
| 1 ☐ | 2 ☐ | 3 ☐ | 4 ☐ | 5 ☐ | 6 ☐ | 7 ☐ | 8 ☐ | 9 ☐ | 10 ☐ | ☐ |

**3 How certain are you that you are able to successfully urge the patient to expand on his or her problems/worries?**

| Very uncertain | | | | | | | | | Very certain | Not relevant |
|---|---|---|---|---|---|---|---|---|---|---|
| 1 ☐ | 2 ☐ | 3 ☐ | 4 ☐ | 5 ☐ | 6 ☐ | 7 ☐ | 8 ☐ | 9 ☐ | 10 ☐ | ☐ |

**4 How certain are you that you are able to successfully listen attentively without interrupting or changing of focus?**

| Very uncertain | | | | | | | | | Very certain | Not relevant |
|---|---|---|---|---|---|---|---|---|---|---|
| 1 ☐ | 2 ☐ | 3 ☐ | 4 ☐ | 5 ☐ | 6 ☐ | 7 ☐ | 8 ☐ | 9 ☐ | 10 ☐ | ☐ |

**5 How certain are you that you are able to successfully encourage the patient to express thoughts and feelings?**

| Very uncertain | | | | | | | | | Very certain | Not relevant |
|---|---|---|---|---|---|---|---|---|---|---|
| 1 ☐ | 2 ☐ | 3 ☐ | 4 ☐ | 5 ☐ | 6 ☐ | 7 ☐ | 8 ☐ | 9 ☐ | 10 ☐ | ☐ |

| 6 | **How certain are you that you are able to successfully structure the conversation with the patient?** | | | | | | | | | | |
|---|---|---|---|---|---|---|---|---|---|---|---|
| | Very uncertain | | | | | | | | | Very certain | Not relevant |
| | 1 ☐ | 2 ☐ | 3 ☐ | 4 ☐ | 5 ☐ | 6 ☐ | 7 ☐ | 8 ☐ | 9 ☐ | 10 ☐ | ☐ |

| 7 | **How certain are you that you are able to successfully demonstrate appropriate non-verbal behavior (eye contact, facial expression, placement, posture, and voicing)?** | | | | | | | | | | |
|---|---|---|---|---|---|---|---|---|---|---|---|
| | Very uncertain | | | | | | | | | Very certain | Not relevant |
| | 1 ☐ | 2 ☐ | 3 ☐ | 4 ☐ | 5 ☐ | 6 ☐ | 7 ☐ | 8 ☐ | 9 ☐ | 10 ☐ | ☐ |

| 8 | **How certain are you that you are able to successfully show empathy (acknowledge the patient's views and feelings)?** | | | | | | | | | | |
|---|---|---|---|---|---|---|---|---|---|---|---|
| | Very uncertain | | | | | | | | | Very certain | Not relevant |
| | 1 ☐ | 2 ☐ | 3 ☐ | 4 ☐ | 5 ☐ | 6 ☐ | 7 ☐ | 8 ☐ | 9 ☐ | 10 ☐ | ☐ |

| 9 | **How certain are you that you are able to successfully clarify what the patient knows in order to communicate the right amount of information?** | | | | | | | | | | |
|---|---|---|---|---|---|---|---|---|---|---|---|
| | Very uncertain | | | | | | | | | Very certain | Not relevant |
| | 1 ☐ | 2 ☐ | 3 ☐ | 4 ☐ | 5 ☐ | 6 ☐ | 7 ☐ | 8 ☐ | 9 ☐ | 10 ☐ | ☐ |

| 10 | **How certain are you that you are able to successfully check patient's understanding of the information given?** | | | | | | | | | | |
|---|---|---|---|---|---|---|---|---|---|---|---|
| | Very uncertain | | | | | | | | | Very certain | Not relevant |
| | 1 ☐ | 2 ☐ | 3 ☐ | 4 ☐ | 5 ☐ | 6 ☐ | 7 ☐ | 8 ☐ | 9 ☐ | 10 ☐ | ☐ |

| 11 | **How certain are you that you are able to successfully make a plan based on shared decisions between you and the patient?** | | | | | | | | | | |
|---|---|---|---|---|---|---|---|---|---|---|---|
| | Very uncertain | | | | | | | | | Very certain | Not relevant |
| | 1 ☐ | 2 ☐ | 3 ☐ | 4 ☐ | 5 ☐ | 6 ☐ | 7 ☐ | 8 ☐ | 9 ☐ | 10 ☐ | ☐ |

| 12 | How certain are you that you are able to successfully close the conversation by assuring, that the patient's questions have been answered? | | | | | | | | | |
|---|---|---|---|---|---|---|---|---|---|---|
| Very uncertain | | | | | | | | | Very certain | Not relevant |
| 1 | 2 | 3 | 4 | 5 | 6 | 7 | 8 | 9 | 10 | |
| ☐ | ☐ | ☐ | ☐ | ☐ | ☐ | ☐ | ☐ | ☐ | ☐ | ☐ |

## In your daily work with patients/relatives

**The next five questions concern specific conversation situations with patients/relatives. Please answer every question, and only use the 'not relevant' box if the question asked does not apply to you in your daily work.**

| 13 | How certain are you that you are able to successfully cope with emotional patients/relatives? | | | | | | | | | |
|---|---|---|---|---|---|---|---|---|---|---|
| Very uncertain | | | | | | | | | Very certain | Not relevant |
| 1 | 2 | 3 | 4 | 5 | 6 | 7 | 8 | 9 | 10 | |
| ☐ | ☐ | ☐ | ☐ | ☐ | ☐ | ☐ | ☐ | ☐ | ☐ | ☐ |

| 14 | How certain are you that you are able to successfully handle angry patients/relatives? | | | | | | | | | |
|---|---|---|---|---|---|---|---|---|---|---|
| Very uncertain | | | | | | | | | Very certain | Not relevant |
| 1 | 2 | 3 | 4 | 5 | 6 | 7 | 8 | 9 | 10 | |
| ☐ | ☐ | ☐ | ☐ | ☐ | ☐ | ☐ | ☐ | ☐ | ☐ | ☐ |

| 15 | How certain are you that you are able to successfully communicate difficult news to patients/relatives? | | | | | | | | | |
|---|---|---|---|---|---|---|---|---|---|---|
| Very uncertain | | | | | | | | | Very certain | Not relevant |
| 1 | 2 | 3 | 4 | 5 | 6 | 7 | 8 | 9 | 10 | |
| ☐ | ☐ | ☐ | ☐ | ☐ | ☐ | ☐ | ☐ | ☐ | ☐ | ☐ |

| 16 | How certain are you that you are able to successfully manage your time with patients/relatives? | | | | | | | | | |
|---|---|---|---|---|---|---|---|---|---|---|
| Very uncertain | | | | | | | | | Very certain | Not relevant |
| 1 | 2 | 3 | 4 | 5 | 6 | 7 | 8 | 9 | 10 | |
| ☐ | ☐ | ☐ | ☐ | ☐ | ☐ | ☐ | ☐ | ☐ | ☐ | ☐ |

| 17 | How certain are you that you are able to successfully involve the patients/relatives in the decisions being made? | | | | | | | | | | |
|---|---|---|---|---|---|---|---|---|---|---|---|
| | Very uncertain | | | | | | | | | Very certain | Not relevant |
| | 1 ☐ | 2 ☐ | 3 ☐ | 4 ☐ | 5 ☐ | 6 ☐ | 7 ☐ | 8 ☐ | 9 ☐ | 10 ☐ | ☐ |

# Background data

**The following questions concern you and your employment. Please answer every question, and only use the 'not relevant' box if the question asked does not apply to you in your daily work.**

| 18 | **What is your gender?** | Female ☐ | Male ☐ |
|---|---|---|---|

| 19 | **What year were you born?** | ____________________ |
|---|---|---|

| 20 | **What language is spoken at home?** | Danish ☐ | Danish and other language ☐ | Other language ☐ |
|---|---|---|---|---|

| 21 | **What is your profession?** | | | | |
|---|---|---|---|---|---|
| | Physician ☐ | Nurse ☐ | Assistant ☐ | Therapist ☐ | Other ☐ Please specify:________________ |

| 22 | **How long have you been employed in your current position?** | | | | | |
|---|---|---|---|---|---|---|
| | Less than ½ year ☐ | ½ year – less than 1 year ☐ | 1 year – less than 2 years ☐ | 2 years – less than 5 years ☐ | 5 years – less than 10 years ☐ | More than 10 years ☐ |

| 23 | **Have you participated in any communication skills training courses after completing your education?** | | |
|---|---|---|---|
| | Yes ☐ | No ☐ | Don't know ☐ |

**If you have anything to add, or wish to elaborate any of your answers, please list below:**

**Thank you for completing the questionnaire!**
